# Unique Presentation of Syphilis With Ocular Involvement: A Case Report

**DOI:** 10.7759/cureus.38793

**Published:** 2023-05-09

**Authors:** Gagandeep Dhillon, Ripudaman S Munjal, Harpreet Grewal, Pranjal Sharma, Rahul Kashyap

**Affiliations:** 1 Internal Medicine, Baltimore Washington Medical Center (BWMC), Glen Burnie, USA; 2 Nephrology, St. Joseph Medical Center, Stockton, USA; 3 Radiology, Florida State University College of Medicine, Pensacola, USA; 4 Nephrology, Northeast Ohio Medical University, Rootstown, USA; 5 Medicine, Drexel University College of Medicine, Philadelphia, USA; 6 Global Clinical Scholars Research Training (GCSRT), Harvard Medical School, Boston, USA; 7 Critical Care Medicine, Mayo Clinic, Rochester, USA

**Keywords:** uveitis, csf, penicillin, syphilis, ocular syphilis, neurosyphilis

## Abstract

Ocular syphilis could be the first sign of undiagnosed syphilis. In addition to otosyphilis, it can be observed in the primary, secondary, or tertiary stages of syphilis. Nonspecific clinical symptoms make diagnosis difficult. We report a patient who presented with generalized weakness and blurry vision for the past four to five days. In this case, we emphasize the importance of repeated cerebrospinal fluid (CSF) examinations as they led to the diagnosis of ocular syphilis and appropriate neurosyphilis treatment. It must be suspected in patients with primary or secondary neurological symptoms, such as blurred vision and weakness. Treponema, the causative organism, is invisible under light microscopy and is mostly identified by its distinct spiral movements under darkfield microscopy. Once the diagnosis was made, the patient was started on penicillin treatment to prevent spread to the brain and dorsal spinal cord. The patient responded well to antibiotic treatment, with improvement in visual acuity, and was discharged with close neurological and ophthalmological follow-up.

## Introduction

Ocular syphilis can occur at any stage of syphilis and can involve various structures of the eye. The most common clinical manifestations are posterior uveitis and panuveitis [1]. Other ocular manifestations include retinal vasculitis, anterior uveitis, interstitial keratitis, and optic neuropathy. It can also lead to a decrease in visual acuity and blindness. Early diagnosis, ophthalmological evaluation, and treatment initiation are vital for managing the clinical symptoms and sequelae of ocular syphilis. Urgent ophthalmological consultations are recommended for patients with positive syphilis serology results and ocular complaints. Lumbar puncture (LP) is recommended for patients with ocular or neurological complications of syphilis. LP can aid in the diagnosis of ocular syphilis [2]. It should be managed according to treatment guidelines for neurosyphilis. Here, we discuss the case of a 68-year-old male who presented to our hospital with generalized weakness and blurry vision.

## Case presentation

A 68-year-old male with a medical history (MH) of hypertension and hyperlipidemia presented to the hospital emergency department with complaints of weakness and blurry vision for the last four to five days. He also reported pain in the left eye and photophobia. No other significant MH was reported; however, he did report the presence of frequent oral ulcers over the last few years.

Upon evaluation, the patient was hemodynamically stable. Neither focal neurological deficits nor meningeal signs were noted. An ocular examination performed in the emergency room revealed bilateral papillary edema. Considering his symptoms, the patient underwent a computed tomography head scan, with no remarkable findings reported. He was admitted to the medical service with a plan to have a neurology consult. 

The next day (hospital day two), the patient underwent a cerebrospinal fluid (CSF) examination via LP by a resident team, which was an inadequate sample with minimal fluid collection. In addition, the opening pressure was not measured; however, proteins and glucose in the fluid were within normal limits. At that time, there was no suspicion for a mass lesion in the cranial cavity. The patient subsequently underwent non-contrast magnetic resonance imaging (MRI) of the brain, which was also unremarkable. The neurology team was consulted, and the patient was started on prednisolone (60 mg) as per their recommendation.

Additional laboratory results were obtained, including a hepatitis panel and HIV testing, both of which were negative. Despite being administered 60 mg of prednisolone for a few days, his symptoms in general did not improve, including weakness and ocular findings. Thus, ophthalmology was consulted on day three. After the patient was evaluated by ophthalmology, neurology was reconsulted on day four, and on their advice, a serum fluorescent treponemal antibody absorption test (FTA-ABS) was performed, which was positive. Neurology also recommended a repeat LP by interventional radiology. A repeat LP revealed a normal opening pressure. However, the measurement of treponema in the CSF test was positive. A definitive diagnosis of ocular syphilis (neurosyphilis) was made on day five. The patient was treated as an inpatient with conventional penicillin antibiotics for 14 days. The patient responded well to antibiotic treatment, with improvement in visual acuity, and was discharged with close neurological and ophthalmological follow-up.

## Discussion

Over the last few years, there has been a notable increase in syphilis cases. The diagnosis of neurosyphilis can be challenging because of its nonspecific clinical symptoms. There is still debate regarding whether ocular involvement can be considered neurosyphilis. Syphilis can be diagnosed using the venereal disease research laboratory and rapid plasma regain (RPR) tests, which quantify the amount of serum anticardiolipin antibodies [3,4]. Treponema is a small organism that is invisible under light microscopy and is mostly identified by its distinct spiral movements under dark-field microscopy. *Treponema pallidum*-specific tests such as FTA-ABS, *T. pallidum* particle agglutination (TPPA), and *T. pallidum* hemagglutination assay measure the amount of serum antibodies against approximal antigens [5].

The patient had presented with weakness and blurred vision for several weeks. He also reported oral ulcers over the last few years. Considering his symptoms, the initial suspicion was an acute neurological process versus an immunological disease, based on previously published literature [6]. However, after neurological and ophthalmological evaluations, clinical suspicion played a major role, and a decision was made to repeat LP in our presented case.

Ocular syphilis should be treated similarly to neurosyphilis. For neurosyphilis, the conventional regimen consists of 12-24 million units of intravenous benzylpenicillin Q4 hours for 10-21 days. Ceftriaxone 1-2 g daily for 10-14 days could be considered for patients with a penicillin allergy with close monitoring for cross-reactivity. Doxycycline can be an effective treatment, but it has not been approved by the CDC or European guidelines for the treatment of neurosyphilis.

There are a few case reports published similar to our case [7]. The patient presented with painless vision loss but also had signs of meningeal irritation, photophobia, and Kernig’s sign. With the increase in syphilis cases in the US, more complications are expected. In this case, vision loss or meningeal involvement was not observed.

A six-year case series of ocular syphilis showed interesting results [8]. With positive TPPA and RPR results, as well as an elevated white blood cell count and protein quantification in the CSF test, LP is important to conduct even though it is not mandatory in diagnosing ocular syphilis. These were important in choosing a standard neurosyphilis treatment protocol rather than only intramuscular penicillin therapy. This was similar to our case, in which the patient received a standard neurosyphilis treatment protocol. Syphilis should be considered in the differential for patients with primary or secondary neurological symptoms, such as blurred vision and weakness. It can mimic auto-immune diseases like giant cell arteritis [9]. It is even more important to have a high level of suspicion given the increased prevalence in the urban underserved community in the United States [10]. 

## Conclusions

In our case of a patient with ocular symptoms of syphilis, it was important to repeat the CSF examination as it led to a diagnosis of ocular syphilis and appropriate neurosyphilis treatment. Syphilis should be considered in the differential for patients with primary or secondary neurological symptoms, such as blurred vision and weakness. These patients should be started immediately on treatment to prevent further invasion of *Treponema pallidum* spirochetes into the brain and dorsal column of the spinal cord.
